# Surgical management of tethered spinal cord syndrome through biportal endoscopic approach: a novel technical note

**DOI:** 10.3171/2024.1.FOCVID23228

**Published:** 2024-04-01

**Authors:** Mehmet İlker Özer, Mehmet Can Ezgü, Ali Kaplan, Zeliha Çulcu Gürcan, Oğuz Kağan Demirtaş

**Affiliations:** 1 Neurosurgery Department of Sincan Research and Training Hospital, Ankara; and; 2 University of Health Sciences, Gulhane Education and Research Hospital, Department of Neurosurgery, Ankara, Turkey

**Keywords:** tethered cord release, minimally invasive surgery, biportal endoscopic approach, filum terminale separation, lumbar spine surgery

## Abstract

This video article explores a case of tethered cord release through a minimally invasive biportal endoscopic approach. A 24-year-old female with chronic back pain and thigh numbness underwent surgery. The chosen approach involved biportal endoscopic technique, demonstrating precision with minimal bone excision. Preoperative imaging revealed a midline fusion defect at L5 and abnormal conus medullaris termination. The surgical procedure involved one-sided hemilaminectomy, durotomy, and careful filum terminale separation. Postoperatively, radiological exams confirmed success with minimal bone defect. Emphasizing minimal invasiveness, reduced bone excision, and muscle sparing, this technique showcased successful outcomes, enabling the patient’s rapid postoperative recovery without complications.

The video can be found here: https://stream.cadmore.media/r10.3171/2024.1.FOCVID23228

## Transcript

In this video article, we will examine a case of tethered cord release using a minimally invasive spinal surgery technique called biportal endoscopic approach,^1^ which has become increasingly popular in recent years.^2–9^

### 0:34 Case Presentation.

Our patient is a 24-year-old female who has been experiencing severe back pain radiating to the buttocks with episodic attacks for the past 8 years. She occasionally reports numbness in her inner thighs. There are no active complaints related to urinary or fecal incontinence.

During the physical examination, no motor deficits were observed, but there is mild hypoesthesia in the sacral dermatomes. Deep tendon reflexes are normal, and inspection reveals increased fat accumulation and hairiness in the sacral region disproportionate to the patient’s weight.

### 1:07 Preoperative Radiography and CT.

Preoperative radiological examinations revealed a midline fusion defect at the L5 level.

### 1:14 Preoperative MRI Study.

On MRI examination, the conus medullaris terminates at the L3 level, and a short, thick, and fatty filum terminale with a hyperintense appearance is noted on the T1 sequence.

### 1:26 Triangulation Phase.

For the surgical approach, a classic unilateral biportal endoscopic approach was chosen. Triangulation was performed at the left L5 spinolaminar junction. A 3-mm cranial incision was made at the level of the L5 pedicle to create a viewing port, and a trocar was introduced through this port. A 1-cm skin incision was made at the level of the left S1 pedicle to create a working port, and a working channel was established using thickened dilators. The dilators in the working port were removed, and the surgery proceeded through the free skin.

### 2:03 Triangulation Check.

After completing the triangulation phase, level control was confirmed with lateral and anteroposterior fluoroscopy images.

### 2:12 Anatomical Orientation and One-Sided Hemilaminectomy.

After the verification of the triangulation level with fluoroscopy, saline irrigation is started and landmarks such as lamina/facet are defined. Starting from the left L5 spinolaminar junction, a minimal partial laminectomy was performed using a spherical arthroscopic burr and Kerrison rongeur.

### 2:34 Flavectomy.

The ligamentum flavum was dissected from the dura with a nerve hook. Then flavectomy was performed with a Kerrison.

### 2:52 Separation of ATA Ligament.

Since the level is L5–S1, the ATA ligament^10^ in the midline is separated from the dura with sharp dissection to avoid irregular dural tear.

### 3:06 Durotomy.

Durotomy is started from the midline with a scalpel, then approximately 8-mm durotomy completed with a microscissors.

### 3:31 Dura Hanging.

Both edges of the durotomy hanged with 8-0 Prolene sutures. Since our approach is unilateral, the purpose of suturing the contralateral dura leaf is not to increase exposure. By elevating both dural leaves, we reduced the migration of the sacral rootlets toward the extradural space and prevented them from interfering with the filum terminale dissection.

### 4:01 Electrophysiological Study.

Filum terminale and rootlets are stimulated through electrophysiological study. Working in saline here did not interfere with neuromonitoring.

Firstly, we stimulated neural tissue and the electrophysiological study proved that it was the sacral rootlet which innervates anal sphincter. Then we stimulated the filum at three different locations and there was no response at the neuromonitor.

### 4:25 Coagulation of the Filum Terminale.

The vascular structures of the filum terminale are coagulated using bipolar electrocautery, and the filum is cut with microscissors.

### 4:55 Detethering the Filum Terminale.

The neural tissues adhered to the filum terminale are dissected from it with a microdissector and microhook. The single remaining vascular structure is coagulated with bipolar and then severed. It is observed that the free ends of the filum move away from each other in both cranial and caudal directions.

### 5:15 Closing.

The rootlets herniated outside the dura are carefully reinserted into the dura with a dissector at the end of the operation. Then the approximately 2-hour-long surgical procedure terminated.

As we planned a minimal durotomy (6–8 mm) and utilized the advantages of the endoscopic technique, there was no muscle dissection and consequently no potential dead space. This approach eliminated the expectation of complications such as pseudomeningocele and CSF fistula. Consequently, dural repair was deemed unnecessary.

### 5:44 Postoperative MRI Study.

Postoperative radiological examination confirmed the separation of the cranial and caudal ends of the filum terminale on T1-weighted MR images.

### 5:53 Postoperative CT Study.

On postoperative CT examination, a minimal bone defect is observed at the left L5 spinolaminar junction. The major advantage of this approach is the successful execution of the surgery with minimal bone excision without the need for total laminectomy.

### 6:09 Outcome.

In conclusion, the patient was mobilized 24 hours after surgery, and there were no cerebrospinal fluid fistula or neurological deficits in the postoperative period. The patient was discharged 72 hours after surgery.

The patient’s low-back pain, which affected her daily life, showed a dramatic improvement in the early postoperative period.

In the postoperative control examination, the patient described that she could hold her urine for a longer period of time and urinate a higher volume at a time. Therefore, it was evaluated that the patient had an unrecognized urinary dysfunction, which was improved postoperatively.

The postoperative 1st month MRI showed no evidence of pseudomeningocele and CSF fistula.

## Disclosures

The authors report no conflict of interest concerning the materials or methods used in this study or the findings specified in this publication.

## Author Contributions

Primary surgeon: Demirtaş, Özer. Assistant surgeon: Kaplan, Çulcu Gürcan. Editing and drafting the video and abstract: Demirtaş, Özer, Ezgü, Kaplan. Critically revising the work: Demirtaş, Özer, Ezgü, Kaplan. Reviewed submitted version of the work: all authors. Approved the final version of the work on behalf of all authors: Demirtaş. Supervision: Demirtaş, Kaplan.

## Supplemental Information

### Patient Informed Consent

The necessary patient informed consent was obtained in this study.
